# Protective Effect of Blackcurrant on Liver Cell Membrane of Rats Intoxicated with Ethanol

**DOI:** 10.1007/s00232-012-9429-3

**Published:** 2012-04-20

**Authors:** Barbara Szachowicz-Petelska, Izabela Dobrzyńska, Elżbieta Skrzydlewska, Zbigniew Figaszewski

**Affiliations:** 1 Institute of Chemistry, University in Białystok, Al. Piłsudskiego 11/4, 15-443 Białystok, Poland; 2 Department of Analytical Chemistry, Medical University of Białystok, Mickiewicza 2, 15-230 Białystok, Poland; 3 Laboratory of Electrochemical Power Sources, Faculty of Chemistry, University of Warsaw, Pasteur St. 1, 02-093 Warsaw, Poland

**Keywords:** Amino acids, Blackcurrant, Ethanol, Liver cell membrane proteins, Peptides, Phospholipids, Surface charge density

## Abstract

Chronic ethanol intoxication oxidative stress participates in the development of many diseases. Nutrition and the interaction of food nutrients with ethanol metabolism may modulate alcohol toxicity. One such compound is blackcurrant, which also has antioxidant abilities. We investigated the effect of blackcurrant as an antioxidant on the composition and electrical charge of liver cell membranes in ethanol-intoxicated rats. Qualitative and quantitative phospholipid composition and the presence of integral membrane proteins were determined by high-performance liquid chromatography. Electrophoresis was used to determine the surface charge density of the rat liver cell membranes. Ethanol intoxication is characterized by changes in cell metabolism that alter the structure and function of cell membrane components. Ethanol increased phospholipid levels and altered the level of integral proteins as determined by decreased phenylalanine, cysteine, and lysine. Ethanol significantly enhanced changes in the surface charge density of the liver cell membranes. Administration of blackcurrant to rats intoxicated with ethanol significantly protected lipids and proteins against oxidative modifications. It is possible that the beneficial effect of blackcurrant is connected with its abilities to scavenge free radicals and to chelate metal ions.

Several exterior factors could be implicated in the oxidative stress formation and cell injury, for example, chronic consumption of ethanol that induces reactive oxygen species (ROS) production (Ponappa and Rubin 2000; Vallet et al. 1997).

The oxidative stress induced by chronic ethanol consumption has been implicated in changes in the structure and function of liver cell components, including membrane phospholipids and proteins. Any perturbation in the action of the cell is manifested by variations in the action of the cell membrane, i.e., in its electric double layer (Szachowicz-Petelska et al. 2010; Dobrzyńska et al. 2010). An essential property of the electric double layer is its electric charge, which can be altered by various xenobiotics or by metabolic transformations.

Ethanol is rapidly absorbed from the gastrointestinal tract, and about 90 % of it is metabolized in the liver. There, ethanol is oxidized into acetaldehyde and then into acetate. These processes are accompanied by free radical generation (Ponappa and Rubin 2000; Vallet et al. 1997). Acetaldehyde and ROS can react with amino acids, peptides, and proteins, modifying their composition and function (Grimsrud et al. 2008; Dorn and Petersen 2002). ROS can also react with lipids causing peroxidation (Kato et al. 1990). Free radical peroxidation, especially of unsaturated lipids, disrupts the important structural and protective functions associated with biomembranes. Certain in vivo pathological events result from this oxidation (Wagner et al. 1994). Therefore, alcohol abuse has been related to a number of biochemical changes and disorders in humans and animals (Ward et al. 2009).

Nutrition and the interaction of food nutrients with ethanol metabolism may modulate alcohol toxicity. Diets rich in fruits are associated with a reduced risk for this pathology, and protection has often been attributed to antioxidant vitamins such as vitamin C, vitamin E, and β-carotene. Although fruits are primary sources for these nutrient antioxidant, other dietary components may also be important protective agents. Flavonoids are plant polyphenolic compounds ubiquitous in fruits. Flavonoids are primarily categorized into flavonols, flavones, flavanols, flavanones, and anthocyanins (Prior 2003). Anthocyanins are the largest group of phenolics in blackcurrants, and they have shown a high antioxidant activity, which influences the oxidation of low-density lipoprotein and food lipids (Satué-Gracia et al. 1997; Fukumoto and Mazza 2000; Smith et al. 2000). Blackcurrants, which are rich in anthocyanins and other phenolics, have been found to posses a varying antioxidant ability (Fukumoto and Mazza 2000; Kähkönen et al. 2001; Moyer et al. 2002).

Therefore, we investigated whether blackcurrant consumption affects the phospholipid and integral membrane protein content as well as the electrical properties of liver cell membranes of ethanol-intoxicated rats.

## Materials and Methods

Blackcurrant (*Ribes nigrum* L.) was used as a juice (containing 28 % pure blackcurrant juice). Blackcurrant juice was purchased in the local supermarket. Rats drank this commercial blackcurrant juice ad libitum instead of water. The content of drinking vessels was renewed every day with 250 ml of fresh juice. The total amount of phenols in the juice was spectrophotometrically determined by using Folin–Ciocatleu’s phenol reagent (Kapasakalidis et al. 2006). The total amount of polyphenols in blackcurrant juice was 1,269 mg of gallic acid equivalents per liter. Anthocyanin concentration was determined by high-performance liquid chromatography (HPLC) with a diode-array detector; detection was at 520 nm (Kapasakalidis et al. 2006). The concentration of four main anthocyanins in blackcurrant juice was 18.28, 14.06, 2.33, and 1.61 μmol/l for delphinidin-3-rutinoside, cyaniding-3-rutinoside, delphinidin-3-glucoside, and cyanidnin-3-glucoside, respectively. The level of vitamin C in the juice was determined by HPLC with a UV detector (Ivanović et al. 1999). The concentration of vitamin C was 50.03 mg/l.

### Animals

Twelve-month-old male Wistar rats were used for the experiment. They were housed in groups with free access to a granular standard diet and water and maintained under a normal light–dark cycle. All experiments were approved by the local ethics committee in Białystok, Poland, according to the Polish Act Protecting Animals of 1997.

The animals were divided into the following groups. The control group was treated intragastrically with 1.8 ml of physiological saline every day for 4 weeks (*n* = 6). The blackcurrant group received blackcurrant juice ad libitum instead of water for 1 week. Next, animals in this group were treated intragastrically with 1.8 ml of physiological saline and received black tea solution ad libitum instead of water every day for 4 weeks (*n* = 6). The ethanol group was treated intragastrically with 1.8 ml of ethanol in doses from 2.0 to 6.0 g/kg body weight every day for 4 weeks. The dose of ethanol was gradually increased by 0.5 g/kg body weight every 3 days (*n* = 6).

The ethanol and blackcurrant groups were given blackcurrant juice ad libitum instead of water for 1 week. Next the animals were treated intragastrically with 1.8 ml of ethanol in doses from 2.0 to 6.0 g/kg body weight and received blackcurrant juice ad libitum instead of water every day for 4 weeks.

### Isolation of Liver Cell Membranes

Livers (approximately 1.5 g) were homogenized in a solution containing 1 mM NaHCO_3_ (pH 7.6) and 0.5 mM CaCl_2_ in a loose-fitting Dounce homogenizer. The addition of 0.5 mM CaCl_2_ increased the cell membrane sedimentation, as determined by measurement of 5′-nucleotidase activity (Ipata 1967). Membrane fragments were separated from nuclei and mitochondria by rate-zonal centrifugation of the low-speed pellet, as described previously (Evans 1970). The sediment was homogenized in sucrose (1.22 g/cm^3^ density) and in the next step was covered with sucrose (1.16 g/cm^3^ density). The cell membranes were separated by centrifugation at 2,000×*g* for 25–35 min. Membrane purity was determined by spectrophotometric determination of 5′-nucleotidase (EC 3.1.3.5) activity as described previously (Ipata 1967).

### Isolation and Analysis of Phospholipids by HPLC

The Folch method was used to extract phospholipids (Folch et al. 1957). The cell membrane was homogenized in a chloroform–methanol mixture (2:1 volume ratio). The solution was then filtered with degreased paper filters, and the precipitate was washed with an extracting solution (8:4:3 chloroform:methanol:aqueous calcium chloride solution 0.05 M calcium chloride). The suspension was centrifuged at 500×*g* for 2 min, the organic and the aqueous phases were separated, and the aqueous phase was shaken again with a chloroform, methanol, and water mixture (3:48:47 volume ratio), and the phases were separated. The organic phases were combined and evaporated to dryness. The extract was dissolved in 200 μl of hexane:isopropanol mixture (3:2) (Ostrowska et al. 2000). Addition of 0.03 % tert-butylhydroxytoluene and flushing with nitrogen at each step in the procedure were used to prevent oxidation during lipid extraction.

HPLC analysis was performed on the extracted phospholipids to assess the quantities phosphatidylinositol (PI), phosphatidylserine (PS), phosphatidylethanolamine (PE), and phosphatidylcholine (PC). The isolated phospholipids were separated by group analysis in a silica gel column using normal phase (NP) HPLC; acetonitrile–methanol–phosphoric acid (85 %) mixture (130:5:1.5 volume ratio) by isocratic elution at 1 ml/s flow rate and 214 nm wavelength (Dobrzyńska et al. 2005b).

### Extraction of Membrane Proteins

The liver cell membranes were homogenized in 5 mM NaOH. PMSF (phenyl–methyl–sulfonyl fluoride) was added to a final concentration of 1 μM to inhibit proteolysis. The suspension was centrifuged for 45 min at 1,000×*g* (Josić et al. 1985).

The residual cell membranes were solubilized in 30 ml buffer containing 20 mM Tris/HCl (pH 7.4) and 1 % Triton X-100 at 4 °C. The suspension was centrifuged at 1,000×*g* for 10 min. The supernatant was incubated at 32 °C for 2 h (Tani et al. 1997) and was then dialyzed against distilled water and evaporated until dry.

### Trypsin Hydrolysis of Proteins

The protein extract was weighed and dissolved in phosphate buffer (pH 7.4) to yield a final protein concentration of approximately 0.10 mg/ml. A stock solution of trypsin (0.05 mg/ml in H_2_O) was added at an enzyme:substrate ratio of 1:25. The reaction mixtures were incubated at 37 °C for 1 h. Hydrolysis was stopped by the addition of PMSF to a final concentration of 1 μM) (Persaud et al. 2000), and the hydrolysate was then evaporated until dry and dissolved in 200 μl H_2_O.

### Separation of the Peptide Mixture of Integral Membrane Proteins by HPLC

**Fig. 1:**
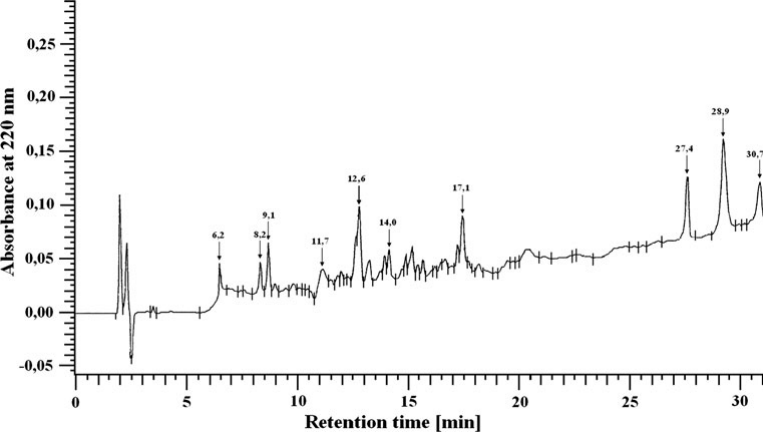
Typical separation of peptides from liver integral membrane proteins (UV detected at 220 nm)

After hydrolysis, the peptides were separated by HPLC on a LichroCART RP-18 column 100A (5 μm, 250 × 4.0 mm) equilibrated with solvent A (0.1 % trifluoroacetic acid (TFA) in H_2_O) and eluted with a linear gradient to 20 % solvent B (0.1 % TFA in acetonitrile) during the first 8 min, to 70 % solvent B during the next 20 min, and to 100 % solvent B during the final 4 min at 220 nm. The flow rate was 1 ml/min (Skrzydlewska et al. 1998). The Merck HPLC system was equipped with a pump, a UV detector, an analog interface module D-6000 A, and System Manager software. A typical separation of the peptide mixture containing liver integral membrane proteins is provided in Fig. 1.

### Peptide Assignment

**Fig. 2:**
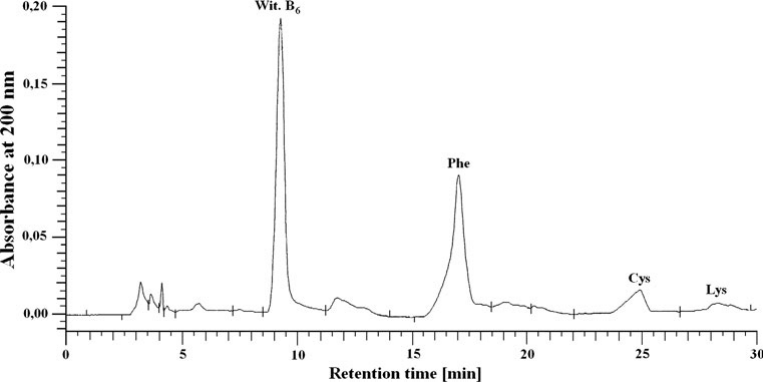
Chromatogram for the amino acids Phe, Cys, and Lys, which occurred in the all of the peptides for each treatment group after hydrolysis of liver cell membrane protein isolates (UV detected at 200 nm)

The amino acid compositions of isolated peptides (6.2, 8.2, 9.1, 11.7, 12.6, 14, 17.1, 27.4, 28.9, and 30.7 min) were determined by HPLC after acid hydrolysis under vacuum in the presence of 6 M HCl for 24 h at 110 °C. The amino acid separation was performed on a Lichrosorb NH_2_ column 100A (5 μm, 250 × 4.6 mm). The mobile phase consisted of solvents A (0.01 M KH_2_PO_4_, pH 4.3) and B (a 500:70 mixture of acetonitrile/water). All separations were performed with a 5–50 % gradient of solvent A using a flow rate of 1 ml/min. The amino acids were detected at a wavelength of 200 nm (Schuster 1980). All the peptides originated from different groups that consistently contained the following three amino acids: phenylalanine (Phe), cysteine (Cys), and lysine (Lys). Figure 2 shows the separation of these amino acids from a typical peptide mixture after the hydrolysis of proteins isolated from liver cell membranes.

Under the chromatographic conditions tested, a linear relationship was verified in the ranges 20–80 μg/ml for Phe, 0.15–1.0 μg/ml for Cys, and 250–400 mg/ml for Lys using standardized solutions and analysis of variance of the regression (*r* ^2^). The *r* ^2^ values for all of these compounds were 0.990.

### Electrochemical Methods

To determine the surface charge density of the cell membrane, liver tissue was exposed to trypsin action. Received cells were put into the measuring vessel, and then electrophoretic mobility on dependent pH was measured with a Zetasizer Nano ZS (Malvern Instruments, UK).

The surface charge density has been determined by the equation σ = ηu/d, where u is the electrophoretic mobility, η is the viscosity of the solution, and d is the diffuse layer thickness (Krysiński and Tien 1986). The diffuse layer thickness was determined by a formula from Barrow (1996):

$$ d = \sqrt {\frac{{\varepsilon \varepsilon _{0} RT}}{{2F^{2} I}}}$$

where *R* is the gas constant, *T* is the temperature, *F* is the Faraday number, *I* is the ionic strength of 0.9 % NaCl, and ε and ε_o_ are the relative and absolute permittivities of the medium.

Acidic (C_TA_) and basic (C_TB_) functional group concentrations and their average association constants with hydrogen (K_AH_) or hydroxyl (K_BOH_) ions were determined as described previously (Dobrzyńska et al. 2005a).

### Statistical Analysis

Data are expressed as mean ± standard deviation. These data were analyzed by standard statistical techniques, specifically one-way analysis of variance with Tukey’s test for multiple comparisons, to determine significant differences between different groups. The data were analyzed separately for treatment groups. A *P* value of less than 0.05 was considered statistically significant.

## Results

### Blackcurrant Administration Affects the Phospholipid Composition of Liver Cell Membranes from Ethanol-Intoxicated Rats

**Fig. 3:**
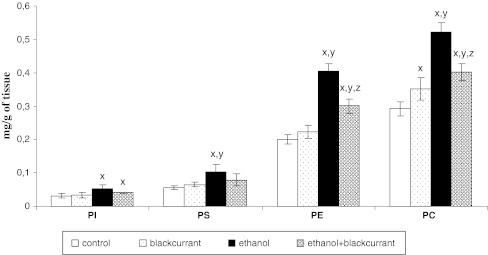
Blackcurrant affects the liver cell membrane content of four phospholipid classes. After exposure to ethanol, blackcurrant, both, or neither, rat liver cell membranes were isolated and the phospholipid content assessed as described in “Materials and Methods” section. Data points represent mean ± SD, *n* = 6 (^*x*^ *P* < 0.05 in comparison with values for control group; ^*y*^ *P* < 0.05 in comparison with values for blackcurrant group; ^*z*^ *P* < 0.05 in comparison with values for ethanol group)

Ethanol intoxication caused an increase in the phospholipid content in the liver cell membrane compared with the control group (Fig. 3). The content of the individual phospholipids—phosphatidylinositol (PI), phosphatidylserine (PS), phosphatidylethanolamine (PE), and phosphatidylcholine (PC)—increased by about 60, 90, 100, and 80 %, respectively. Administration of alcohol to blackcurrant-exposed rats caused a smaller increase in PI, PS, PE, and PC than administration of ethanol alone. In the rats treated with blackcurrant alone and the control rats, no essential differences were observed in the phospholipid content.

### Blackcurrant Administration Affects Integral Membrane Proteins of Liver Cell Membranes from Ethanol-Intoxicated Rats

Selective hydrolysis of integral membrane proteins to peptides, resolution by HPLC, and subsequent determination of the amount of specific amino acids within individual peptides is one method used to study pathological changes in integral membrane proteins. Up to a 40 % decrease in integral membrane protein levels was observed in the ethanol group relative to the control and blackcurrant groups (Table 1). In contrast, the group that consumed both blackcurrant and ethanol had integral membrane protein levels that were higher than the ethanol group but lower than the control and blackcurrant-alone groups.

**Table 1:** Effect of blackcurrant on integral membrane protein content in liver cell membranes of rats receiving ethanol, blackcurrant, or both

Group	Integral protein (mg/g tissue)
Control	4.4 ± 0.35
Blackcurrant	4.0 ± 0.34
Ethanol	2.81 ± 0.22^*x*,*y*^
Ethanol + blackcurrant	3.52 ± 0.28^*x*,*y*,*z*^

Data points represent mean ± SD, *n* = 6

^*x*^ *P* < 0.05 in comparison with values for control group

^*y*^ *P* < 0.05 in comparison with values for blackcurrant group

^*z*^ *P* < 0.05 in comparison with values for ethanol group

**Fig. 4:**
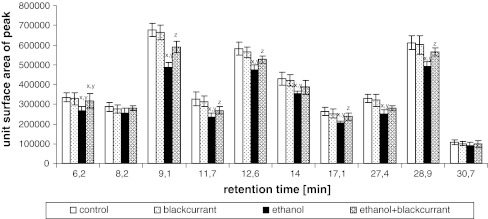
Blackcurrant affects the peptide content of hydrolyzed liver cell membranes from rats fed ethanol. Data points represent mean ± SD, *n* = 6 (^*x*^ *P* < 0.05 in comparison with values for control group; ^*y*^ *P* < 0.05 in comparison with values for blackcurrant group; ^*z*^ *P* < 0.05 in comparison with values for ethanol group)

Figure 4 shows changes in peptide content after hydrolysis of proteins isolated from liver cell membranes of the control, ethanol, blackcurrant, and ethanol with blackcurrant groups. Ethanol intoxication caused an decrease in peptide levels in the ethanol group relative to the control, blackcurrant, and ethanol with blackcurrant groups. The peptide contents at the retention times of 6.2, 8.2, 9.1, 11.7, 12.6, 14, 17.1, 27.4, 28.9, and 30.7 min decreased approximately 20, 10, 30, 30, 20, 20, 25, 25, 20, and 15 %, respectively, in the ethanol group relative to the control group. Rats that consumed blackcurrant with ethanol had higher integral membrane protein content than those that consumed ethanol alone. No significant differences were observed between the control group and the blackcurrant-alone group.

**Fig. 5:**
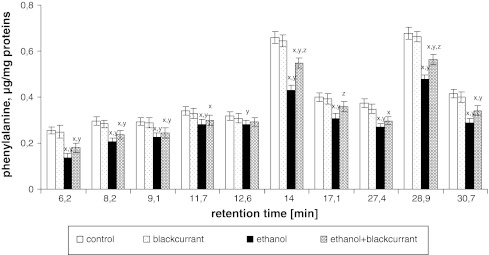
Blackcurrant affects Phe levels in integral membrane proteins from liver cell membranes. Data points represent mean ± SD, *n* = 6 (^*x*^ *P* < 0.05 in comparison with values for control group; ^*y*^ *P* < 0.05 in comparison with values for blackcurrant group; ^*z*^ *P* < 0.05 in comparison with values for ethanol group)

**Fig. 6:**
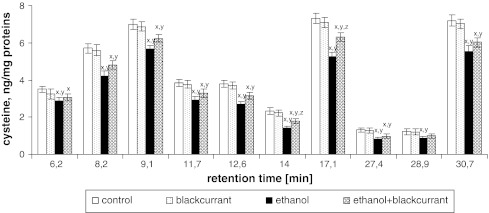
Blackcurrant affects Cys levels in integral membrane proteins from liver cell membranes. Data points represent mean ± SD, *n* = 6 (^*x*^ *P* < 0.05 in comparison with values for control group; ^*y*^ *P* < 0.05 in comparison with values for blackcurrant group; ^*z*^ *P* < 0.05 in comparison with values for ethanol group)

**Fig. 7:**
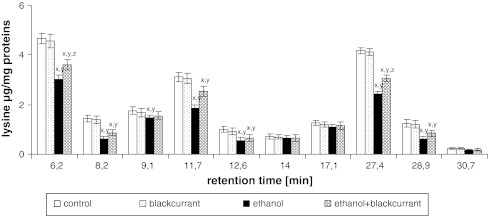
Blackcurrant affects Lys levels in integral membrane proteins from liver cell membranes. Data points represent mean ± SD, *n* = 6 (^*x*^ *P* < 0.05 in comparison with values for control group; ^*y*^ *P* < 0.05 in comparison with values for blackcurrant group; ^*z*^ *P* < 0.05 in comparison with values for ethanol group)

No significant differences in the content of Phe, Cys, and Lys individually were observed in rats treated with blackcurrant relative to the control group. The ethanol group showed a decrease in the amount of individual amino acids relative to the control group (Figs. 5, 6, 7). The amino acid contents at the retention times 6.2, 8.2, 9.1, 11.7, 12.6, 14, 17.1, 27.4, 28.9, and 30.7 min decreased by approximately 45, 30, 20, 20, 10, 35, 20, 30, 30, and 15 %, respectively for Phe; decreased by approximately 20, 30, 20, 20, 30, 40, 30, 40, 30, and 20 %, respectively, for Cys; and decreased by approximately 35, 60, 20, 40, 50, 10, 10, 40, 50, and 30 %, respectively, for Lys. The largest decrease was seen with Lys, which was reduced by 60 % (8.2 min), 40 % (11.7 min), 50 % (12.6 min), 40 % (27.4 min), and 50 % (28.9 min). Administration of alcohol to rats consuming blackcurrant led to an increase in the amount of Phe, Cys, and Lys relative to the ethanol-alone group.

### Effect of Blackcurrant on Electric Properties of Liver Cell Membrane of Ethanol-Intoxicated Rats

Administering ethanol to the rats provokes an increase in liver cell membrane C_TA_ (26 %) and C_TB_ (31 %) compared with the control group (Table 2). The concentration of acidic (11 %) and basic (10 %) groups in the liver cell membrane decreased in animals fed ethanol and blackcurrant compared with animals fed ethanol only. Administering blackcurrant to the rats increased the association constant of the acidic groups (K_AH_) of the liver cell membrane compared to the control groups. In both groups, K_BOH_, the association constant of the basic groups of the liver cell membrane, decreased after administering blackcurrant compared with the control group. Administering ethanol induces a decrease in K_AH_ (49 %) and an increase in K_BOH_ (48 %) in the rat liver cell membranes compared with the control groups. Administering blackcurrant with ethanol also induced an increase in the K_AH_ value (15 %) and a decrease in the K_BOH_ value (45 %) compared with the ethanol groups.

**Table 2:** Effect of blackcurrant on the C_TA_, C_TB_, K_AH_, and K_BOH_ of liver cell membranes from ethanol-intoxicated rats

Group	C_TA_ (10^−7^−mol/m^2^)	C_TB_ (10^−7^ mol/m^2^)	K_AH_ (m^3^/mol)	K_BOH_ (10^6^ m^3^/mol)
Control	2.31 ± 0.08	0.96 ± 0.08	34.04 ± 1.10	7.10 ± 0.21
Blackcurrant	2.28 ± 0.07	0.95 ± 0.07	33.54 ± 1.01	7.55 ± 0.20^*x*^
Ethanol	2.92 ± 0.10^*x*,*y*^	1.28 ± 0.06^*x*,*y*^	22.81 ± 1.27^*x*,*y*^	10.51 ± 0.24^*x*,*y*^
Ethanol + blackcurrant	2.62 ± 0.09^*x*,*y*,*z*^	1.17 ± 0.05^*x*,*y*,*z*^	27.12 ± 1.11^*x*,*y*,*z*^	8.90 ± 0.25^*x*,*y*,*z*^

Data points represent mean ± SD, *n* = 6

^*x*^ *P* < 0.05 in comparison with values for control group

^*y*^ *P* < 0.05 in comparison with values for blackcurrant group

^*z*^ *P* < 0.05 in comparison with values for ethanol group

## Discussion

The liver is the main organ responsible for metabolism of both endogenous and exogenous compounds, and therefore it is also one of the first target organs for the toxic action of xenobiotics or their reactive metabolites.

Ethanol intoxication causes a wide variety of metabolic disorders in human and animals, mainly caused by free radicals, and hydroxyl radicals in particular. Hydroxyl radicals readily react with cell components, especially with lipids and proteins (Gieseg et al. 2000). This is manifested by an increase in lipid peroxidation and protein oxidative modification products observed in this study. Oxidative modifications of proteins are usually initiated by hydroxyl radicals, as a result of which oxidation of amino acid residues and oxidation in polypeptide chain of protein take place that lead to fragmentation and/or formation of cross-link bindings (Davies 1987; Stefek et al. 2005; Wu et al. 2009). All amino acids are susceptible to attack by free radicals, although some of them are more vulnerable than others—those that are most sensitive to oxidation include aromatic amino acids such as phenylalanine and tyrosine. Moreover, the levels of bistyrosine—the product of a reaction between free radicals and tyrosine—are known to increase during ethanol ingestion (Łuczaj et al. 2006). As such, bistyrosine production is a useful marker for protein modification by hydroxyl radicals (Giulivi et al. 2003). As a sulfydryl amino acid, Cys is also extremely sensitive to free radicals. Reports have shown that the cysteine:cystine ratio of proteins is altered under oxidizing conditions (Kalyanaraman 1995). The occurrence of these types of reactions would explain the decrease in the number of Phe and Cys amino acids detected in our study (Figs. 5, 6).

Ethanol-induced oxidative modifications of membrane cell phospholipids were also observed in this and earlier studies. This was manifested by an increase in the level of all phospholipids in liver cell membranes (Fig. 3).

**Fig. 8:**
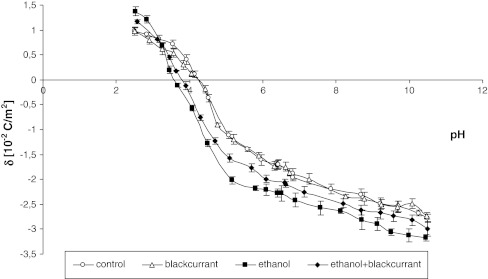
Blackcurrant prevents changes in electrical properties of liver cell membranes from rats fed ethanol

Therefore, the increase in phospholipids caused by ethanol intoxication is accompanied by decreased integral membrane proteins in liver cell membranes. Changes in membrane composition are connected with changes in cell membrane charge. Our results demonstrate that the electrical properties of liver cell membranes are affected by ethanol intoxication (Fig. 8; Table 2). An increase in the amount of specific phospholipids results in the appearance of additional functional groups, both positively and negatively charged, at the membrane surface. Changes in membrane structure caused by the modification of protein structure lead to yield higher negative electric charge at high pH values and lower positive electric charges at low pH values (Szachowicz-Petelska et al. 2005, 2008; Dobrzyńska et al. 2008). Variations in the number and kind of functional groups result in changes in C_TA_ and C_TB_ and, in turn, in their association constant values (Table 2).

Oxidative stress and oxidative damage of cell components caused by ethanol are also counteracted by compounds that have antioxidant properties, which are able to modulate ethanol metabolism in the organism. One of such potent antioxidant is blackcurrant, which is known as a fruit with a strong in vitro antioxidative capacity, but its in vivo antioxidant efficacy has not yet been characterized (Salobir et al. 2010; Viljanen et al. 2004; Valentova et al. 2007). In this study, it has been shown that blackcurrant significantly protects phospholipids as well as protein against oxidative modification of Phe, Cys, and Lys.

Blackcurrant is rich in monomeric and polymeric phenolic compounds providing protection toward both lipid and protein oxidation (Viljanen et al. 2004). Phenolic compounds have been shown to be effective antioxidants in inhibiting lipid oxidation (Kähkönen et al. 2001; Mullen et al. 2002; Kähkönen and Heinonen 2003) as well as potent radical scavengers (Riedl and Hagerman 2001). In addition, anthocyanins have been shown to chelate metal ions at moderate pH with their ionized hydroxyl groups of the B ring (Prior 2003). It was postulated that phenolic compounds inhibit oxidation of proteins both by retarding the oxidation reactions by binding to the proteins and by forming complexes between protein molecules (Viljanen et al. 2005).

Our results provide evidence for the effectiveness of blackcurrant in the prevention of ethanol-induced oxidative stress. This indicates the possibility of the use of this natural antioxidant in preventing disorders initiated by oxidative stress.
